# Supplementary material for: Evolutionary design of explainable algorithms for biomedical image segmentation
Source: Nat Commun. 2023 Nov 6;14:7112. doi: 10.1038/s41467-023-42664-x (PMC10628266; doi:10.1038/s41467-023-42664-x)
Supplement: Supplementary file 1 — Supplementary Information [file 41467_2023_42664_MOESM1_ESM.pdf]

Supplementary Figures & Tables

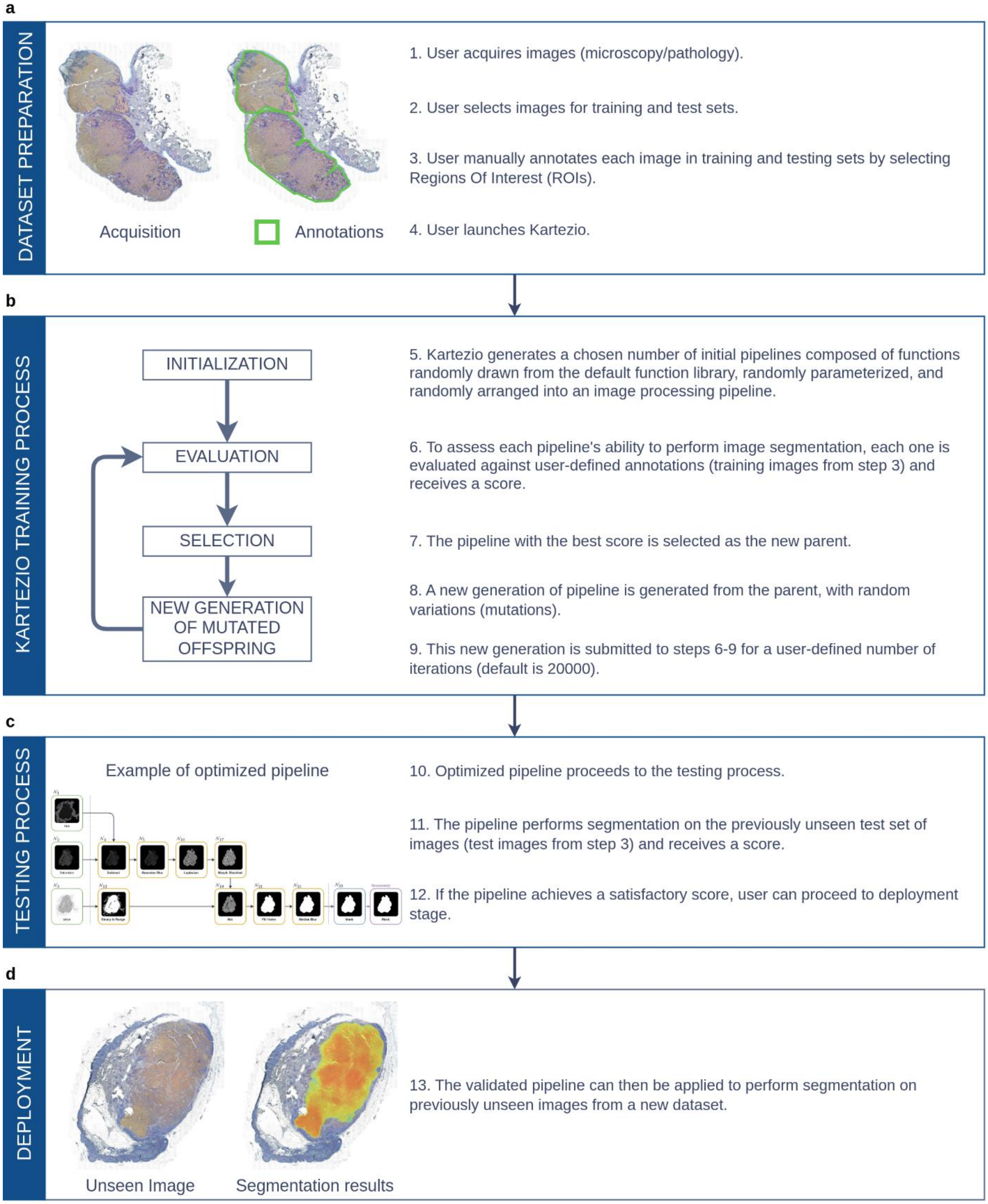

Supplementary Fig. 1 | Conceptual summary of Kartezio

(a) Dataset preparation by experimentalist; **(b)** illustration of Kartezio training; **(c)** illustration of Kartezio testing; **(d)** deployment of Kartezio-generated pipeline.

Supplementary Figures & Tables

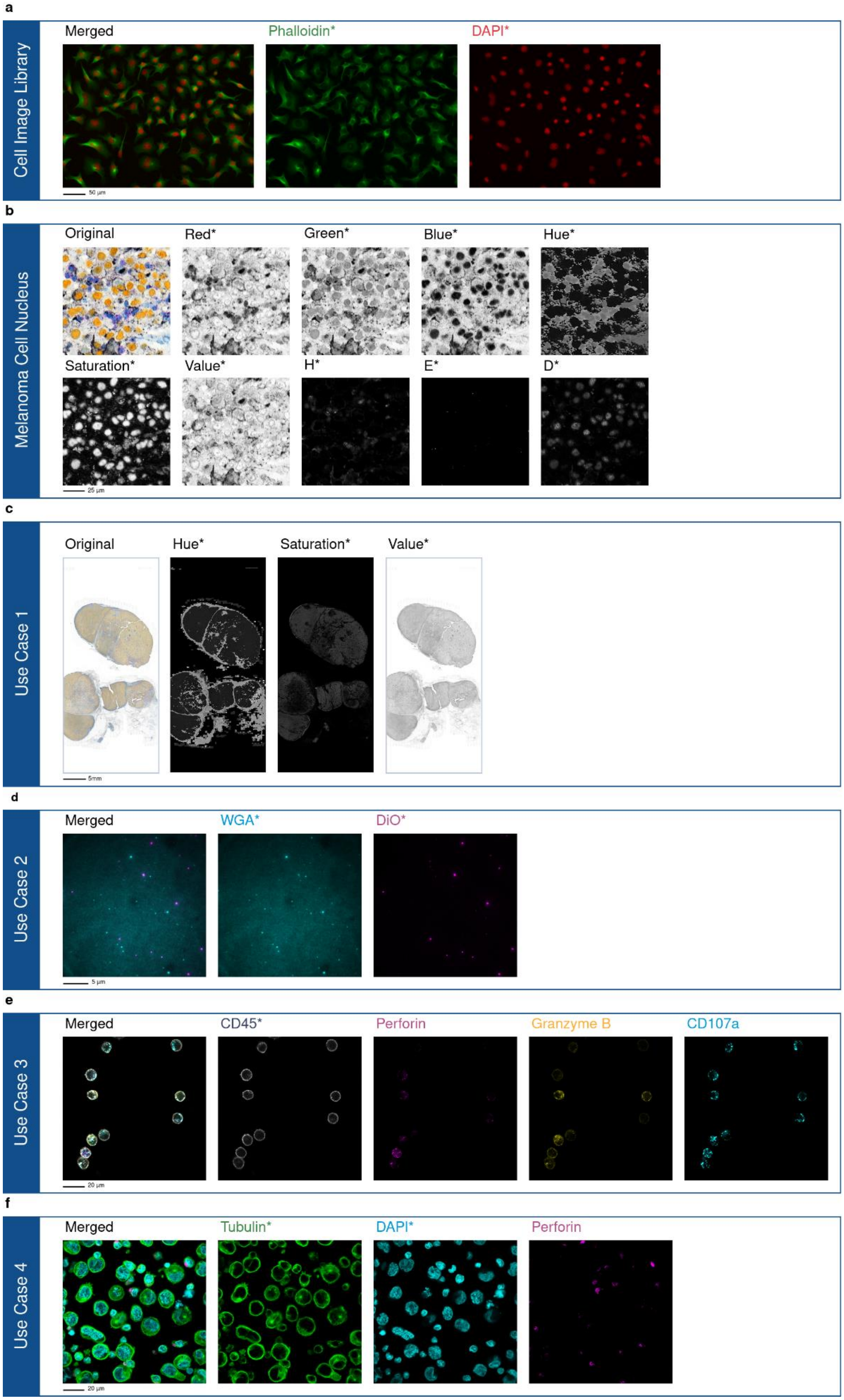

Supplementary Figures & Tables

Supplementary Fig. 2 | Illustrative images from each Use Case

(a-f) Representative images from each Use Case. Total numbers of images in the training and test datasets for each Use Case are summarized in **Supplementary Table 3**; numbers of *in silico* experiments performed for each Use Case (i.e. number of "runs") are shown in **Supplementary Table 4**. **(a)** Images from the Cellpose dataset (derived from Cell Image Library [23, 28], reproduced with permission, and described in **Fig. 2**) wherein *in vitro* neurons were stained with phalloidin (*green*) and DAPI (*red*). Original images available from: Weimiao Yu, Lee H.K., Hariharan, S., Bu W.Y., Ahmed, S. CIL_40217, Mus musculus, Neuroblastoma. http://cellimagelibrary.org/images/40217. (2012). **(b, c)** Images from the melanoma cohort [29] comprised of fixed tissue slices stained for Sox10 (*orange*), CD8 (*purple*), CD107a (*black*) and counterstained with hematoxylin (*blue*). **(b)** Original high resolution images (evaluated in **Fig. 2**) were split into RGB, HSV or HED color spaces as shown. **(c)** Original low resolution images (described in **Fig. 3**) were split into HSV color space as shown. **(d)** Extracellular particles derived from polyclonal CTLs (shown in **Fig. 4**) were stained with WGA (*cyan*) and DiO (*magenta*) and imaged using TIRFM. **(e)** Human polyclonal $CD8^+$ CTLs (shown in **Fig. 5**) were stained with antibodies directed against CD45 (*gray*), perforin (*magenta*), granzyme B (*yellow*) and CD107a (*cyan*). **(f)** A mixed population of cells containing both human clonal CTLs and target cells **(Fig. 6)** was stained with DAPI (*cyan*) and antibodies directed against α-tubulin (*green*) and perforin (*magenta*). * indicates channel was used as a model input for the corresponding Use Case.

Supplementary Figures & Tables

```python
class ModelMelanoma(CodeModel):
    def __init__(self):
        super().__init__(endpoint=EndpointWatershed())


    def _parse(self, x):
        x_2 = x[1]
        x_3 = x[2]
        node_4 = self.call_node("bitwise_and", [x_3, x_2], [159, 219])
        node_5 = self.call_node("open", [node_4], [57, 55])
        node_6 = self.call_node("inrange", [node_5], [139, 253])
        node_7 = self.call_node("threshold", [node_5], [88, 119])
        z_1 = node_7
        z_2 = node_6
        Z = [z_1, z_2]
        return Z
```

Supplementary Fig. 3 | Automatically generated Python classes by Kartezio from given genotype

The Python class automatically generated from the evolved genotype illustrated in **Fig. 1** and utilized in **Fig. 2d.** Source code image generated using the carbon tool available at carbon.now.sh.

Supplementary Figures & Tables

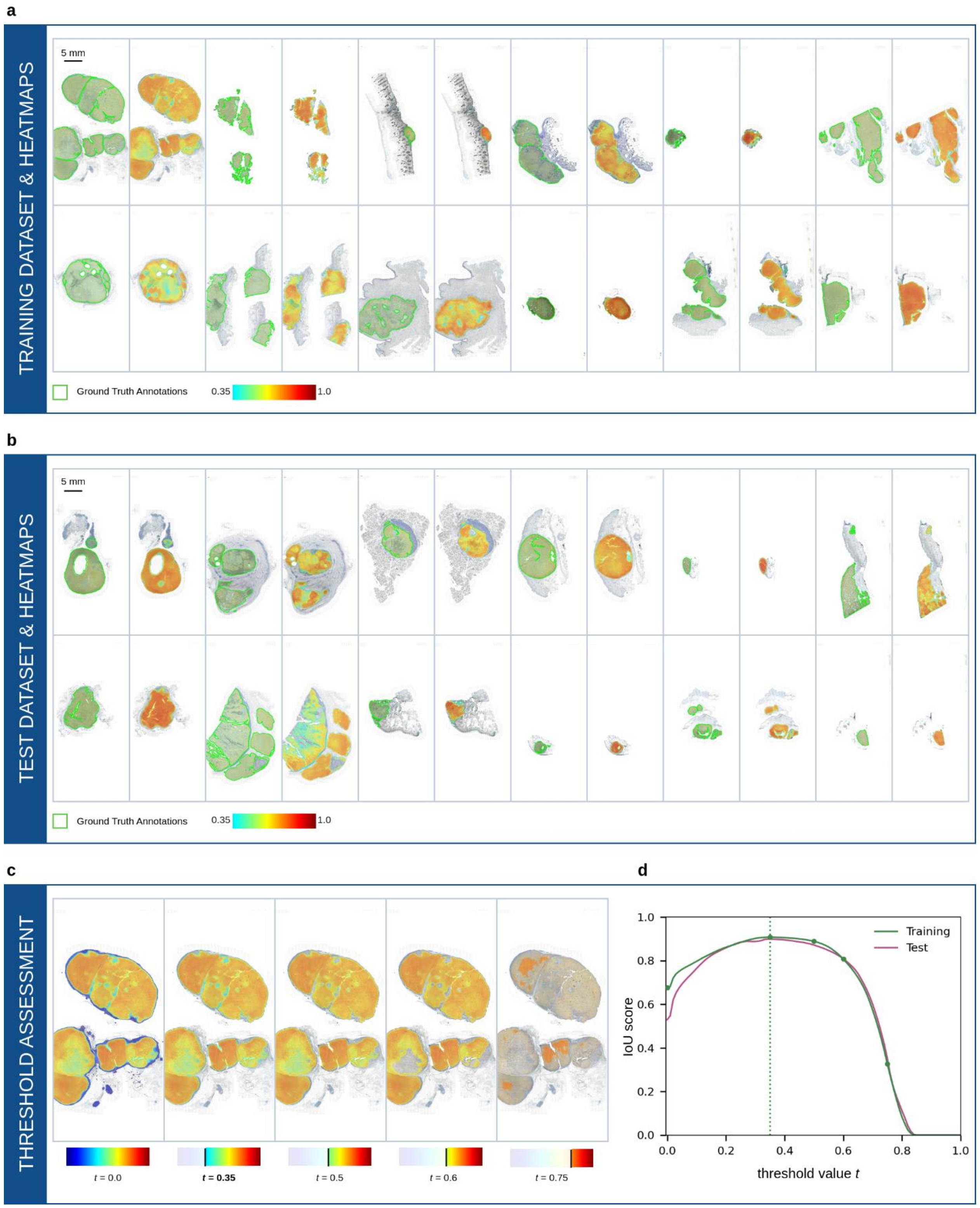

Supplementary Figures & Tables

Supplementary Fig. 4 | Tumor nodule datasets and heatmap threshold analysis

Use Case 1: **a, b)** Tumor nodule datasets comprised of fixed tissue slices stained for Sox10 (*orange*), CD8 (*purple*), CD107a (*black*) and counterstained with hematoxylin (*blue*) were used for training **(a)** and testing **(b)** of Kartezio-generated semantic segmentation pipelines (n=100 models). Left panels in each pair depict tumor annotations as delineated by an expert pathologist (*green lines*). Right panels depict the predictions of 100 Kartezio-generated models whose outputs have been normalized and averaged to generate probability-based heatmaps of predicted tumor contours (minimum probability depicted is 0.35; maximum is 1.0). **(c)** A representative histology image with heatmap overlay and threshold set to different values (t = 0, 0.35, 0.5, 0.6, and 0.7) **(d)** Fitness (IoU score) of the model ensemble containing 100 Kartezio-generated pipelines as a function of threshold value (with increments of 0.01) for the training dataset (12 histopathology images, *green*) and testing dataset (12 histopathology images, *magenta*). Dotted green line indicates the optimal threshold value (t = 0.35) as determined using the training dataset.

Supplementary Figures & Tables

Supplementary Table 1 | Functions included in the default library L accessible by Kartezio

Function	Index	Symbol	Arity	Parameters	Sources	Domain
max	0	MAX	2	0	OpenCV	u8
min	1	MIN	2	0	OpenCV	u8
mean	2	MEAN	2	0	OpenCV	u8
add	3	ADD	2	0	OpenCV	u8
subtract	4	SUB	2	0	OpenCV	u8
bitwise_not	5	NOT	1	0	OpenCV	u8
bitwise_or	6	BOR	2	0	OpenCV	u8
bitwise_and	7	BAND	2	0	OpenCV	u8
bitwise_mask	8	ANDM	2	0	OpenCV	u8
bitwise_xor	9	BXOR	2	0	OpenCV	u8
sqrt	10	SQRT	1	0	OpenCV	f32
pow2	11	POW	1	0	OpenCV	f32
exp	12	EXP	1	0	OpenCV	f32
log	13	LOG	1	0	Numpy	u8
median_blur	14	BLRM	1	1	OpenCV	u8
gaussian_blur	15	BLRG	1	1	OpenCV	u8
laplacian	16	LPLC	1	0	OpenCV	f64
sobel	17	SOBL	1	2	OpenCV	f64
robert_cross	18	RBRT	1	1	OpenCV	f64
canny	19	CANY	1	2	OpenCV	u8
sharpen	20	SHRP	1	0	OpenCV	u8
gabor	21	GABR	1	2	OpenCV	u8
abs_diff	22	ABSD	1	2	OpenCV	u8
abs_diff2	23	ABS2	2	0	OpenCV	u8
fluo_tophat	24	FLUO	1	2	Handmade	f32
rel_diff	25	RELD	1	1	Handmade	f32
erode	26	EROD	1	2	OpenCV	u8
dilate	27	DILT	1	2	OpenCV	u8
open	28	OPEN	1	2	OpenCV	u8
close	29	CLSE	1	2	OpenCV	u8
morph_gradient	30	MGRD	1	2	OpenCV	u8
morph_tophat	31	MTHT	1	2	OpenCV	u8
morph_blackhat	32	MBHT	1	2	OpenCV	u8
fill_holes	33	FILL	1	0	Handmade	u8
rm_small_objects	34	RMSO	1	1	Skimage	bool
rm_small_holes	35	RMSH	1	1	Skimage	bool
threshold	36	TRH	1	2	OpenCV	u8
threshold_at_1	37	TRH1	1	1	OpenCV	u8
dt	38	DTRF	1	1	OpenCV	u8
dt_and_thresh	39	DTTR	1	2	OpenCV	u8
inrange_bin	40	BRNG	1	2	OpenCV	u8
inrange	41	RNG	1	2	OpenCV	u8

Supplementary Figures & Tables

Supplementary Table 2 | Fitness scores achieved by Kartezio with descending numbers of training images

Training Images	Median Training ROIs	Training Fitness	Test Images	Test ROIs	Test Fitness
89	4701	**0.853 ± 0.016**	11	521	**0.858 ± 0.020**
80	4168	**0.852 ± 0.029**	11	521	**0.853 ± 0.028**
70	3511	**0.851 ± 0.018**	11	521	**0.852 ± 0.021**
60	3180	**0.858 ± 0.016**	11	521	**0.856 ± 0.019**
50	2629	**0.857 ± 0.015**	11	521	**0.849 ± 0.026**
40	2184	**0.857 ± 0.018**	11	521	**0.850 ± 0.021**
30	1696	**0.861 ± 0.023**	11	521	**0.839 ± 0.027**
25	1306	**0.860 ± 0.021**	11	521	**0.840 ± 0.025**
20	1022	**0.855 ± 0.020**	11	521	**0.843 ± 0.023**
15	771	**0.867 ± 0.028**	11	521	**0.844 ± 0.025**
14	698	**0.864 ± 0.024**	11	521	**0.837 ± 0.032**
13	713	**0.873 ± 0.028**	11	521	**0.832 ± 0.031**
12	632	**0.876 ± 0.028**	11	521	**0.828 ± 0.023**
11	583	**0.865 ± 0.031**	11	521	**0.828 ± 0.036**
10	510	**0.869 ± 0.030**	11	521	**0.825 ± 0.032**
9	451	**0.872 ± 0.031**	11	521	**0.830 ± 0.033**
8	411	**0.888 ± 0.036**	11	521	**0.828 ± 0.029**
7	382	**0.880 ± 0.042**	11	521	**0.807 ± 0.051**
6	351	**0.882 ± 0.031**	11	521	**0.810 ± 0.054**
5	236	**0.886 ± 0.036**	11	521	**0.798 ± 0.061**
4	172	**0.890 ± 0.035**	11	521	**0.790 ± 0.066**
3	160	**0.895 ± 0.044**	11	521	**0.767 ± 0.066**
2	96	**0.918 ± 0.044**	11	521	**0.732 ± 0.102**
1	32	**0.933 ± 0.058**	11	521	**0.584 ± 0.135**

ROI = region of interest; training fitness scores shown represent the mean +/- SD for n = 35 pipelines

Supplementary Figures & Tables

Supplementary Table 3 | Summary of Use Case dataset preparation and analysis

	Use Case 1	Use Case 2		Use Case 3	Use Case 4
Use Case Name	Tumor Nodules	Extracellular Particles (SMAPs)		CTL Lytic Arsenal	Lytic Synapse
Imaging Type	2D IHC	2D IF (TIRF)		3D IF	3D IF
Image Processing Task	Semantic Segmentation	Instance Segmentation		Instance Segmentation	Instance Segmentation
Staining	Sox10, CD8 and CD107a Abs, hematoxylin dye	WGA and DiO probes		CD45, Perforin, Granzyme B and CD107a Abs	α-tubulin and perforin Abs, DAPI probe
Scale	1pixel = 46.8µm	1pixel = 0.107µm		1pixel = 0.264µm	1pixel = 0.264µm
Number of Datasets	1	2		1	1
Dataset Name	Tumor Nodules	WGA Particles	DiO Particles	CTL Cells	CTL/Target Cells
Training Images	12	$1 \xrightarrow{\text{split}} 4$	$1 \xrightarrow{\text{split}} 4$	5	8
Training ROIs	12	56	44	45	262
Testing Images	12	$1 \xrightarrow{\text{split}} 4$	$1 \xrightarrow{\text{split}} 4$	4	4
Testing ROIs	12	48	31	34	150
Post-Segmentation Analysis	Heatmap generation from the average of 100 models	Matching between masks using Intersection Over Union metric followed by feature extraction (MFI, particle size)		Feature extraction from instances (MFI) followed by 2D embedding and visualization using UMAP	Unsupervised machine learning classification using Gaussian Mixture Model followed by synapse detection

IHC = immunohistochemistry; **IF** = immunofluorescence; **CTL** = cytotoxic T lymphocyte; **SMAP** = supra-molecular attack particle; **Abs** = antibodies; **TIRFM** = total internal reflection fluorescence microscopy; **ROI** = region of interest

Supplementary Figures & Tables

Supplementary Table 4 | Summary of Use Case model parameterization

	Dimension	Models	Runs	Name of Model	Model Inputs	Model Outputs	Aggregation	Model Endpoint
Use Case 1	2D	1	100	Nodule Model	$\iota = 3$, {Hue, Sat., Val.}	$o = 1$	-	Threshold
Use Case 2	2D	2	35	WGA Model	$\iota = 1$, {WGA}	$o = 1$	-	Local-Max Watershed
			35	DiO Model	$\iota = 1$, {DiO}	$o = 1$	-	Local-Max Watershed
Use Case 3	3D	1	35	CTL Model	$\iota = 1$, {CD45}	$o = 1$	Average	Local-Max Watershed
Use Case 4	3D	1	35	Cell Model	$\iota = 2$, {DAPI, Tubulin}	$o = 2$	Average	Marker-Controlled Watershed

CTL = cytotoxic T lymphocyte; **WGA** = wheat germ agglutinin; **Sat** = saturation; **Val** = value

Supplementary Figures & Tables

Supplementary Table 5 | Summary of Use Case fitness scores

	Training Min	Training Max	Training Average	Test Min	Test Max	Test Average
Use Case 1	**0.831**	**0.908**	**0.870 ± 0.018**	**0.637**	**0.888**	**0.846 ± 0.047**
Use Case 2 (WGA)	**0.658**	**0.872**	**0.805 ± 0.037**	**0.378**	**0.837**	**0.733 ± 0.083**
Use Case 2 (DiO)	**0.713**	**0.839**	**0.788 ± 0.030**	**0.399**	**0.847**	**0.762 ± 0.090**
Use Case 3	**0.975**	**1.000**	**0.977 ± 0.007**	**0.775**	**0.889**	**0.825 ± 0.028**
Use Case 4	**0.723**	**0.805**	**0.755 ± 0.022**	**0.576**	**0.729**	**0.667 ± 0.034**

WGA = wheat germ agglutinin

Supplementary Figures & Tables

Supplementary Movie

Supplementary Movie 1 | An illustrative example of an instance segmentation pipeline iteratively evolving using Kartezio

This is an illustrative example of an instance segmentation pipeline evolving using Kartezio, according to a 1+ λ evolution strategy wherein $\lambda = 2$. Two training images (denoted *Original Image A* and *Original Image B*) and their corresponding manual annotations (*Annotations A* and *Annotations B*) were selected from the Cellpose specialist dataset originally derived from Cell Image Library (reproduced with permission) [23, 28] and provided to Kartezio, which generated an initial parent pipeline composed of functions randomly drawn from the default function library, randomly parameterized, and randomly arranged into an image processing pipeline. This parent pipeline and two mutated offspring (*Child 1* and *Child 2*) were evaluated against the user-defined annotations for Image A and B. The best pipeline was selected to proceed through the evolutionary selection process, during which the existing parent pipeline was randomly mutated either through changing the functions, the order in which they were arranged in the pipeline, or their parameters. With each iteration (*generation*), this process was repeated. When a new child pipeline outperformed the parent pipeline, it replaced the parent and the evolutionary process continued for a set number of generations (default = 20,000). Of note, in generation 1, all three graphs (parent and two offspring) are randomly generated, while in each subsequent generation, the offspring are generated by mutating the parent pipeline. Original images available from: Weimiao Yu, Lee H.K., Hariharan, S., Bu W.Y., Ahmed, S. CIL_40217, Mus musculus, Neuroblastoma. http://cellimagelibrary.org/images/40217. (2012).

***Supplementary Movie 1** has also been made available online at https://youtu.be/UUW643JEe1Y
