# Supplementary material for: Evolutionary design of explainable algorithms for biomedical image segmentation
Source: Nat Commun. 2023 Nov 6;14:7112. doi: 10.1038/s41467-023-42664-x (PMC10628266; doi:10.1038/s41467-023-42664-x)
Supplement: Supplementary file 3 — Description of Additional Supplementary Files [file 41467_2023_42664_MOESM3_ESM.pdf]

Description of Additional Supplementary Files

File name: Supplementary Movie 1

Description: An illustrative example of an instance segmentation pipeline iteratively evolving using Kartezio. This is an illustrative example of an instance segmentation pipeline evolving using Kartezio, according to a 1+ λ evolution strategy wherein $\lambda = 2$. Two training images (denoted *Original Image A* and *Original Image B*) and their corresponding manual annotations (*Annotations A* and *Annotations B*) were selected from the Cellpose specialist dataset originally derived from Cell Image Library (reproduced with permission) [23, 28] and provided to Kartezio, which generated an initial parent pipeline composed of functions randomly drawn from the default function library, randomly parameterized, and randomly arranged into an image processing pipeline. This parent pipeline and two mutated offspring (*Child 1* and *Child 2*) were evaluated against the user-defined annotations for Image A and B. The best pipeline was selected to proceed through the evolutionary selection process, during which the existing parent pipeline was randomly mutated either through changing the functions, the order in which they were arranged in the pipeline, or their parameters. With each iteration (*generation*), this process was repeated. When a new child pipeline outperformed the parent pipeline, it replaced the parent and the evolutionary process continued for a set number of generations (default = 20,000). Of note, in generation 1, all three graphs (parent and two offspring) are randomly generated, while in each subsequent generation, the offspring are generated by mutating the parent pipeline.

*__Supplementary Movie 1__ has also been made available online at https://youtu.be/UUW643JEe1Y
