# Supplementary material for: Evolutionary design of explainable algorithms for biomedical image segmentation
Source: Nat Commun. 2023 Nov 6;14:7112. doi: 10.1038/s41467-023-42664-x (PMC10628266; doi:10.1038/s41467-023-42664-x)
Supplement: Supplementary file 5 — Reporting Summary [file 41467_2023_42664_MOESM5_ESM.pdf]

nature portfolio

Corresponding author(s): Salvatore Valitutti and Sylvain Cussat-Blanc

Last updated by author(s): Sep 7, 2023

Reporting Summary

Nature Portfolio wishes to improve the reproducibility of the work that we publish. This form provides structure for consistency and transparency in reporting. For further information on Nature Portfolio policies, see our Editorial Policies and the Editorial Policy Checklist.

Statistics

For all statistical analyses, confirm that the following items are present in the figure legend, table legend, main text, or Methods section.

n/a	Confirmed	
☐	☒	The exact sample size (*n*) for each experimental group/condition, given as a discrete number and unit of measurement
☐	☒	A statement on whether measurements were taken from distinct samples or whether the same sample was measured repeatedly
☐	☒	The statistical test(s) used AND whether they are one- or two-sided<br>*Only common tests should be described solely by name; describe more complex techniques in the Methods section.*
☒	☐	A description of all covariates tested
☒	☐	A description of any assumptions or corrections, such as tests of normality and adjustment for multiple comparisons
☐	☒	A full description of the statistical parameters including central tendency (e.g. means) or other basic estimates (e.g. regression coefficient) AND variation (e.g. standard deviation) or associated estimates of uncertainty (e.g. confidence intervals)
☐	☒	For null hypothesis testing, the test statistic (e.g. *F*, *t*, *r*) with confidence intervals, effect sizes, degrees of freedom and *P* value noted<br>*Give P values as exact values whenever suitable.*
☒	☐	For Bayesian analysis, information on the choice of priors and Markov chain Monte Carlo settings
☒	☐	For hierarchical and complex designs, identification of the appropriate level for tests and full reporting of outcomes
☒	☐	Estimates of effect sizes (e.g. Cohen's *d*, Pearson's *r*), indicating how they were calculated

Our web collection on statistics for biologists contains articles on many of the points above.

Software and code

Policy information about availability of computer code

Data collection	Use Case 1: 2D Whole Slide Images acquired by Panoramic 250 Flash II digital microscope (3DHISTECH, Budapest, Hungary).<br>Use Case 2: 2D TIRF Images acquired by Olympus IX83 inverted microscope.<br>Use Case 3: 3D IF Images acquired by LSM 780 (Zeiss) confocal microscope.<br>Use Case 4: 3D IF Images acquired by LSM 780 (Zeiss) confocal microscope.
Data analysis	- Kartezio was developed using Python3.8 and built mainly over Numpy, OpenCV, Scikit-image, and Scikit-learn.<br>- Image handling was performed using ImageJ (1.53o), Zen Black (14.0.27.201) and CellSens (XV 3.26).<br>- Data was visualized using Matplotlib, Seaborn, and OpenCV (Python packages) as well as GraphPad Prism (9.5.0).<br>- Statistical analysis was performed using GraphPad Prism (9.5.0).<br><br>Source code for Kartezio are available for non-commercial use on GitHub (https://github.com/KevinCortacero/Kartezio) with an associated ReadMe file.<br>Kartezio-related scripts specific to this manuscript are also available on GitHub (https://github.com/KevinCortacero/KartezioPaper) with an associated ReadMe file.

For manuscripts utilizing custom algorithms or software that are central to the research but not yet described in published literature, software must be made available to editors and reviewers. We strongly encourage code deposition in a community repository (e.g. GitHub). See the Nature Portfolio guidelines for submitting code & software for further information.

Data

Policy information about availability of data

All manuscripts must include a data availability statement. This statement should provide the following information, where applicable:

- Accession codes, unique identifiers, or web links for publicly available datasets
- A description of any restrictions on data availability
- For clinical datasets or third party data, please ensure that the statement adheres to our policy

All relevant image datasets and genomes associated with this manuscript are archived and accessible online as Source Data files associated with this manuscript. The dataset utilized for comparison with Cellpose is publically available (www.cellpose.org/dataset).

Human research participants

Policy information about studies involving human research participants and Sex and Gender in Research.

Reporting on sex and gender	No individual-level data was provided in this study and thus sex/gender was not reported.
Population characteristics	Use Case 1: The established melanoma clinical cohort was comprised of patients treated for advanced melanoma both at the Oncodermatology Department of the Institut Universitaire du Cancer de Toulouse (IUCT) and at the Centre Hospitalier Universitaire (CHU) de Bordeaux. All histological images were derived from this large anonymized cohort (previously published, Filali et al, Science Advances 2022). No individual-level data was provided in this study.
Recruitment	Use Case 1: Adult patients treated for advanced melanoma both at the Oncodermatology Department of the Institut Universitaire du Cancer de Toulouse (IUCT) and at the Centre Hospitalier Universitaire (CHU) de Bordeaux were included in the study.<br><br>Use Case 2-4: Peripheral blood samples from healthy donors were provided by the Oxford Blood Centre (Research Tissue Bank; UK) or from the Établissement Français du Sang (France) following procedures agreed by the National Institute of Health and French Ministry of the Research respectively. The donors of both blood centers were healthy adults of all ages, eligible to donated blood for transfusion and research purposes, and provided written consent for their blood samples to be used for research. Samples used in this manuscript were randomly selected from the daily pool of donors and sent to research labs as needed. As such, there was no discernible bias in sample selection and peripheral blood samples were fully de-identified prior to being received by research labs. No donor information and no medical records were obtained by the labs.
Ethics oversight	Use Case 1: Samples in Toulouse were stored at the CRB Cancer des Hôpitaux de Toulouse collection. In accordance with French law, this cancer collection was declared to the Ministry of Higher Education and Research (DC-2020-4074) and a transfer agreement was obtained (AC-2020-4031) after approbation by ethical committees. Samples in Bordeaux were stored at the Cancer Biobank of CHU Bordeaux collection. In accordance with French law, this cancer collection was declared to the Ministry of Higher Education and Research (DC 2014-2164) and a transfer agreement was obtained (AC-2019-3595) after approbation by ethical committees.<br><br>Use Case 2: de-identified peripheral blood samples from consenting healthy adult donors were provided by the Oxford Blood Centre (Oxford Radcliffe Biobank, Research Tissue Bank, REC 19/SC/0173), under the Kennedy Institute of Rheumatology ethics agreement number 11-H0711-7, approved by the National Health Service Research Ethics Committee (UK).<br><br>Use Case 3/4: Blood samples were collected and processed following standard ethical procedures after obtaining written informed consent from each donor and approval by the French Ministry of the Research (transfer agreement AC-2020-3971). Approbation by the ethical department of the French Ministry of the Research for the preparation and conservation of cell lines and clones starting from healthy donor human blood samples has been obtained (authorization no. DC-2021-4673).

Note that full information on the approval of the study protocol must also be provided in the manuscript.

Field-specific reporting

Please select the one below that is the best fit for your research. If you are not sure, read the appropriate sections before making your selection.

☒ Life sciences ☐ Behavioural & social sciences ☐ Ecological, evolutionary & environmental sciences

For a reference copy of the document with all sections, see nature.com/documents/nr-reporting-summary-flat.pdf

Life sciences study design

All studies must disclose on these points even when the disclosure is negative.

Sample size	In this computational study, the sample size (n) was the number of models independently generated in each Use Case, with each model representing an independent in silico experiment. Instance segmentation tasks in this manuscript were conducted with a sample size of n=35, which was determined by the number of tasks (one per core) that can be launched simultaneously by the CALMIP supercomputer. This number of independent experiments is quite high (with n=5-10 being standard within the field), but the large sample size was deemed to provide a more robust and useful assessment of Kartezio's performance. Semantic segmentation tasks that are represented as probability heatmaps in the manuscript were the product of n=100 models, in order to increase the robustness of the predictions.
Data exclusions	Images that did not meet the technical quality benchmark for inclusion in the training and test datasets according to expert judgement (e.g. over-saturated images, folded or wrinkled tissue, etc) were not included when these datasets were constructed. No data generated from the training and test datasets were excluded from this manuscript.
Replication	Comparison with gold-standard algorithms: n=35 times for each size of training dataset (1-89 training images)<br>Use Case 1: Algorithm was run n=100 times in parallel as described above.<br>Use Case 2-4: Algorithm was run n=35 times in parallel as described above.
Randomization	In this computational study (which does not include experimental groups per se), sample allocation refers to the allocation of images to the training and test datasets. In the comparison with gold-standard algorithms (Figure 2a,b), the train/test split composition was provided by the original authors (Stringer et al, Nature Methods, 2020); in the current manuscript, training datasets of different sizes (1-89 images) were drawn at random from the 89-image training dataset supplied by Stringer et al. In Use Case 1, images in the training dataset were selected by experts from the patient cohort to reflect the heterogeneity of patient tumors. In Use Case 2-4, train/test split was random.
Blinding	In this computational study, experimental groups per se did not exist and thus blinding was not required. However, in keeping with good practice, human tissue samples were anonymized and thus the expert users generating manual annotations of histology images were blinded to clinical and demographic features of the patients. All experiments performed in silico by AI did not require blinding.

Reporting for specific materials, systems and methods

We require information from authors about some types of materials, experimental systems and methods used in many studies. Here, indicate whether each material, system or method listed is relevant to your study. If you are not sure if a list item applies to your research, read the appropriate section before selecting a response.

Materials & experimental systems

n/a	Involved in the study	
☐	☒	Antibodies
☐	☒	Eukaryotic cell lines
☒	☐	Palaeontology and archaeology
☒	☐	Animals and other organisms
☒	☐	Clinical data
☒	☐	Dual use research of concern

Methods

n/a	Involved in the study	
☒	☐	ChIP-seq
☒	☐	Flow cytometry
☒	☐	MRI-based neuroimaging

Antibodies

Antibodies used

Use Case 1
Anti-CD107a rabbit mAb clone D2D11 #9091 Cell Signaling Technology followed by OmniMap anti-rabbit HRP conjugated secondary antibody (05269679001, ROCHE)
Anti-CD8 IgG1 clone C8/144B M7103 Agilent Technologies followed by OmniMap anti-mouse (05269652001, ROCHE) HRP conjugated secondary antibody
Anti-Sox10 rabbit mAb clone SP267 07560389001, ROCHE followed by OmniMap anti-rabbit HRP conjugated secondary antibody (05269679001, ROCHE)

Use Case 3
Anti-human perforin mAb clone δG9, IgG2b, BD 556434 followed by goat anti-mouse IgG2b AlexaFluor555 Thermofisher A21147
Anti-human CD107a rabbit Ab polyclonal Abcam ab24170 followed by goat anti-rabbit AlexaFluor647 Thermofisher A21245
Anti-human granzyme B mAb clone GB11, IgG1 Thermofisher MA1-80734 followed by goat anti-mouse IgG1 AlexaFluor488 Thermofisher A21121
Anti-human CD45 rat Ab clone YAML501.4 Thermofisher MA5-17687 followed by goat anti-rat AlexaFluor405 Thermofisher A48261

Use Case 4
Anti-human perforin mAb clone δG9, IgG2b, BD 556434 followed by AlexaFluor555 goat anti-mouse IgG2b Thermofisher A21147
Anti-α-tubulin mAb clone DMA1 Sigma Aldrich #T6199 followed by AlexaFluor488 goat anti-mouse IgG1 Thermofisher A21121

Validation	Use Case 1 Anti-CD107a QC Tested for reactivity to human CD107a; recommended for IHC; cited in 375 publications and published by the authors in Filali et al, Science Advances, 2022<br><br>Use Case 1 Anti-CD8 QC Tested for reactivity to human CD8; recommended for IHC; and published by the authors in Filali et al, Science Advances, 2022<br><br>Use Case 1 Anti-Sox10 QC Tested for reactivity to human Sox10 recommended for IHC (dermatopathology); and published by the authors in Filali et al, Science Advances, 2022<br><br>Use Case 3 Anti-human perforin mAb QC Tested for reactivity to human perforin; reported application for IF, published by authors in Khazen et al, Nature Communications, 2015<br><br>Use Case 3 Anti-human CD107a rabbit Ab QC tested for reactivity to human CD107a; used in 571 publications according to manufacturer, published by authors in Khazen et al, Nature Communications, 2015<br><br>Use Case 3 Anti-human granzyme B mAb QC tested for reactivity to human granzyme B; recommended for IF, used in 63 manuscripts and published by authors in Khazen et al, Nature Communications, 2015<br><br>Use Case 3 anti-human CD45 rat Ab QC tested for reactivity to human CD45, recommended for IF, reported in 8 references<br><br>Use Case 4 Anti-human perforin mAb QC Tested for reactivity to human perforin; reported application for IF, published by authors in Khazen et al, Nature Communications, 2015<br><br>Use Case 4 anti-α-tubulin mAb QC Tested for reactivity to human alpha tubulin; recommended for IF

Eukaryotic cell lines

Policy information about cell lines and Sex and Gender in Research

Cell line source(s)	Use Case 2: Polyclonal T cells (donor-derived, UK)<br>Use Case 3: Polyclonal T cells (donor-derived, France)<br>Use Case 4: Antigen-specific clonal T cells (donor-derived, France)<br>JY EBV-tranformed B cell line (obtained from Antonio Lanzavecchia, Basel Institute for Immunology, Switzerland)
Authentication	All cells except JY cells were donor-derived. JY cells were authenticated 09/2020 by Microsynth, Switzerland.<br>Profiling of the human cell lines used highly polymorphic short tandem repeat loci (STRs). STR loci were amplified using the PowerPlex® 16 HS System (Promega). Fragment analysis was done on an ABI3730xl (Life Technologies) and the resulting data were analyzed with GeneMarker HID software (Softgenetics).
Mycoplasma contamination	All cells are tested biweekly for mycoplasma contamination. All cells utilized in this manuscript tested negative for mycoplasma prior to use in experiments.
Commonly misidentified lines<br>(See ICLAC register)	This manuscript does not contain any commonly misidentified lines.
